# Differences in Physical Activity Levels between Healthy and Transplanted Children: Who Needs More Tips?

**DOI:** 10.3390/healthcare11111610

**Published:** 2023-05-31

**Authors:** Eliana Tranchita, Giulia Cafiero, Ugo Giordano, Stefano Palermi, Federica Gentili, Isabella Guzzo, Marco Spada, Federica Morolli, Fabrizio Drago, Attilio Turchetta

**Affiliations:** 1 Sports Medicine Unit, Bambino Gesù Children’s Hospital, Istituto di Ricovero e Cura a Carattere Scientifico, 00165 Rome, Italy; giulia.cafiero@opbg.net (G.C.); ugo.giordano@opbg.net (U.G.); federica.gentili@opbg.net (F.G.); attilio.turchetta@opbg.net (A.T.); 2 Public Health Department, University of Naples Federico II, 80131 Naples, Italy; stefano.palermi@unina.it; 3 Division of Nephrology, Dialysis and Transplantation, Bambino Gesù Children’s Hospital, Istituto di Ricovero e Cura a Carattere Scientifico, 00165 Rome, Italy; isabella.guzzo@opbg.net; 4 Division of Hepato-Bilio-Pancreatic Surgery, Liver and Kidney Transplantation, Bambino Gesù Children’s Hospital, ERN Transplant Child, Istituto di Ricovero e Cura a Carattere Scientifico, 00165 Rome, Italy; marco.spada@opbg.net (M.S.); federica.morolli@opbg.net (F.M.); 5 Paediatric Cardiology and Cardiac Arrhythmias Unit, Bambino Gesù Children’s Hospital, Istituto di Ricovero e Cura a Carattere Scientifico, 00165 Rome, Italy; fabrizio.drago@opbg.net

**Keywords:** physical activity, children, transplant, preventive medicine, sedentary behaviors

## Abstract

Background: Advances in the medical-surgical field have significantly increased the life expectancy of patients undergoing solid organ transplantation but this exposes patients to long-term complications due to chronic therapies and changes in lifestyle. It is known that children affected by pathology tend to be more sedentary and inactivity represents a further risk factor for the onset of non-communicable diseases. The aim of the present study was to compare the lifestyle of two groups of young patients: one group of healthy subjects (HG) and one group of kidney or liver transplant recipients (TG). Methods: Patients were asked to complete Physical Activity Questionnaire for Older Children (PAQ-C). Results: A total of 104 subjects were recruited (50.9% male, mean age 12.8 ± 3.16 years old). No significant differences were observed in the final score between groups when comparing subjects based on health condition (Healthy 2.69 ± 0.65 vs. Transplant Group 2.42 ± 0.88), the intensity of sports activities (Competitive 2.82 ± 0.59 vs. Not Competitive 2.53 ± 0.7) or type of transplant (Liver 2.51 ± 0.91 vs. Kidney 2.16 ± 0.75). Conclusion: The results of this study showed a worrying reality: children are engaged in low levels of physical activity regardless of their health status and in general the level of activity does not reach the recommended values even in the absence of contraindications. So, it is necessary to encourage healthy children to practice more PA and to introduce PA prescriptions for transplanted children to prevent their health from deteriorating due to sedentariness.

## 1. Introduction

In recent decades, more children have had the opportunity to receive solid organ transplants to improve their pathologies, thanks to new achievements in modern medicine. Many centers in Europe and in the world have been created to deal with the transplantation of different types of solid organs and numerous centers have specialized in pediatric transplants, too. The European Liver Transplant Registry (ELTR) reported a total of 146,762 liver transplants performed in Europe until 2016, with approximately 2% performed in children [1]. Similarly, the European Renal Association Registry (ERA) reported that a total of 24,013 kidney transplantations were carried out in 2019 in 34 European-Mediterranean countries, 3% of which involved young patients [2].

The survival rate of Liver Transplant (LT) in children has increased over time and was recently evaluated in a meta-analysis [3], which reviewed a total of 542 titles in the literature. The meta-analysis estimated that the overall survival rate of children after receiving LT is 86.62% after 1 year, 77.74% after 3 years, 73.95% after 5 years, and 68.60% after 10 years from transplantation. The increase in survival rate is due to improvements in organ preservation, surgical techniques, immunosuppression therapies, treatment regimens, and post-operative infection prevention protocols.

Similarly, significant advancements have been made in the lives of patients with kidney disease since the first pediatric kidney transplant was performed in 1982. Therefore, physicians are often called to evaluate the health of these children and provide them with the right indications for their well-being.

Literature data show that children who have undergone organ transplantation develop further comorbidities over time due to the medications used [4] or organ dysfunction. The use of corticosteroids can lead to hypertension, cardiac left ventricular hypertrophy, obesity, dyslipidemia, diabetes, growth, and bone mineralization deficits [4,5,6,7] while the use of immunosuppressive drugs can cause chronic kidney failure, infections, and gastrointestinal diseases and can remarkably increase the risk of cancer onset, such as cancer of the skin/soft tissue, but also lymphoma and leukemia [5,8,9,10]. Having a sedentary lifestyle may aggravate the aforementioned comorbidities and their clinical condition, but fortunately is a modifiable factor. The majority of recipients do not meet the recommended amount of physical activity (PA), resulting in a generally sedentary and inactive lifestyle [11,12,13]. However, a sedentary lifestyle is also becoming typical of the population of healthy young people too [14], especially after the COVID-19 pandemic [15]. Therefore, children are nowadays very far from meeting the amount of PA recommended by guidelines [16] that suggest at least an average of 60 min per day of moderate- to vigorous-intensity, mostly aerobic, physical activity, across the week for children and adolescents. For the same population vigorous-intensity aerobic activities, as well as those that strengthen muscle and bone, are recommended at least 3 days a week.

At the same time, studies in the literature confirm that PA may be a valid instrument to prevent cardiovascular risk factor onset [17], osteoporosis [18], kidney failure, and cancer [19,20]. PA also represents a therapeutic tool that can restore physical, functional, and psychological capacities, and it was demonstrated to be safe, feasible, and effective [21], also in preventing the decline in the quality of life in children who underwent transplantation. Lack of sufficient exercise practice is detrimental to the physical, mental health, and quality of life of transplant recipients while there is robust evidence that exercise can work as a medicine for the health not only of transplanted subjects but also for a variety of neurological, psychiatric, cardiometabolic, respiratory and musculoskeletal pathologies [22]. However, current post-transplant approaches are insufficient to fully address the right amount of physical activity for these patients, and physical exercise is not part of the post-transplant long-term therapy protocols [23]. Therefore, the present study aimed to compare the lifestyle of two groups of young patients. In particular, we analyzed the practice of physical exercise, using validated questionnaires, between children and adolescents belonging to a healthy group (HG) or a transplant recipients’ group (TG).

## 2. Materials and Methods

### 2.1. Study Design

A cross-sectional observational study was carried out over 3 months, at Bambino Gesù Children’s Hospital in Rome, Italy. Patients were asked to complete the “Physical Activity Questionnaire for Older Children (PAQ-C) and Adolescent (PAQ-A)” [24] during the annual sports medicine pre-participation screening and/or during post-transplant follow-up visits, and they voluntarily decided to answer the questions.

The study conforms to the ethical principles of Good Clinical Practice, was conducted according to the Helsinki Declaration and its later amendments, and was approved by the Ethics Committee of Bambino Gesù Children’s Hospital (protocol code 3049_OPBG_2023), following the current Italian regulations. Moreover, all subjects and their parents were verbally informed about the aim and the procedures of the study and signed written informed consent. All participants were assured about the anonymity of data and that the data would have been processed for scientific purposes and in an aggregate manner.

### 2.2. Subjects

Sample size was selected from the total population of children who underwent sports medicine or post-transplant specialistic care. The inclusion criteria were as follows:

1. Aged between 8 and 18 years old;
2. Underwent a solid organ transplant or undergoing to pre-participation screening for sport;
3. At least 2 years elapsed since the kidney/liver transplant;
4. No contraindications to practice sports activities;
5. Stable clinical condition.

The exclusion criteria were as follows:

1. Non-cooperating children for the questionnaire due to age and/or psycho-physical limitations;
2. Patients with a history of congenital heart disease;
3. Patients with other comorbidities or multiple organ transplant;
4. Children who reported having been ill in the previous week;
5. Children with congenital heart disease or other comorbidities were not enrolled to avoid confounding effects of other pathologies on exercise practice.

### 2.3. Questionnaire

All children filled out the PAQs [24] to evaluate the volume of physical activity they practiced. It is a seven-day recall self-administered questionnaire to evaluate the amount of moderate to vigorous physical activity practiced by school-aged children. In particular, PAQs are composed of 10 items, each scoring between 1 (low physical activity) and 5 (high physical activity). The PAQs summary score was calculated taking the average of the results of the items from 1 to 9. Item 10 is not used as part of the summary activity score. These questionnaires were recently validated for the Italian language (PAQ-It) [25]. PAQ-It psychometric properties have been reported as acceptable to good, in particular a good internal consistency (α = 0.82 and 0.84). Scale reliabilities for girls (α = 0.75), boys (α = 0.72), and the total (α = 0.74) were acceptable. Moreover, the PAQ-It total score significantly correlated with total objective moderate to vigorous PA (rho = 0.30), supporting that the questionnaire sufficiently measures the moderate to vigorous PA during a school week [25]. See Table A1.

### 2.4. Statistical Analysis

Data analysis was performed using the R Statistical Software (v. 4.2.2; R Core Team 2021). Descriptive statistics for demographic variables and for questionnaire answers stratifying by groups were computed. In particular, frequency distribution for categorical variables, median and interquartile range, and variance and standard deviation for continuous variables were performed. In order to compare groups for statistical differences, for continuous variables Student’s *t*-Test was used to determine whether the means of two groups were equal to each other, while Pearson’s Chi-squared Test was used to evaluate categorical variables. Results were considered statistically significant for *p*-values ≤ 0.05.

## 3. Results

**Figure 1:**
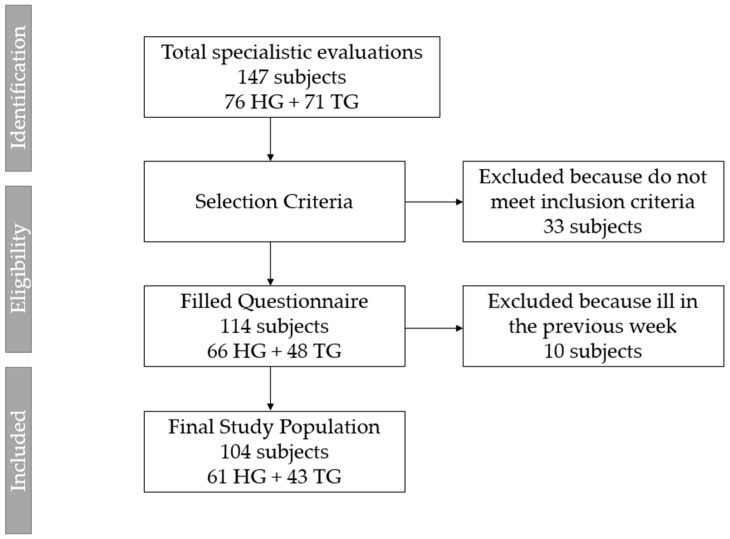
Flowchart of study participants. HG: healthy group; TG: transplant group.

Of the total number of subjects (n = 147) who underwent specialistic evaluation, 33 patients did not meet the inclusion criteria and were discharged. A total of 114 subjects voluntarily filled out the questionnaire, and ten of them were excluded from the analysis because they reported being ill in the previous week, making the data collected through the questionnaire unrepresentative of their daily life. Therefore, data from 104 patients were used for the purpose of the present study (Figure 1).

The demographic characteristics of the study population are shown in Table 1. The mean age of the children was 12.8 ± 3.16 years old (range 8–18) and 50.9% of them were male. Most of them were normal weight (mean 19.84 ± 3.57 kg/m^2^). In the transplant group, approximately 74% of children received liver transplantation and 26% underwent kidney transplantation. Almost half of them received the transplant more than 5 years ago (51%).

The scores of the PAQs questionnaire recorded in our population are reported in Table 2. No significant differences were observed between groups when comparing subjects based on health condition (Healthy vs. Transplant Group), the intensity of sports activities among healthy children (Competitive vs. Not competitive), or type of transplant among recipients (Liver vs. Kidney).

We investigated in detail if there were differences in the score of question 2 regarding the practice of physical education lessons at school, and we found a significant difference between the HG and the TG (*p* = 0.05). However, this difference was not significant when comparing Competitive and Not competitive, nor for the type of transplant (Table 3).

We also investigated if there were differences in the score of question 8 which provide a general impression of physical activity in the last 7 days, and we found a significative difference between the Healthy and the Transplant groups (*p* = 0.02) and between Competitive and Not competitive (*p* = 0.005). The differences were not significant when we compared children based on the type of transplant (Table 4).

**Figure 2:**
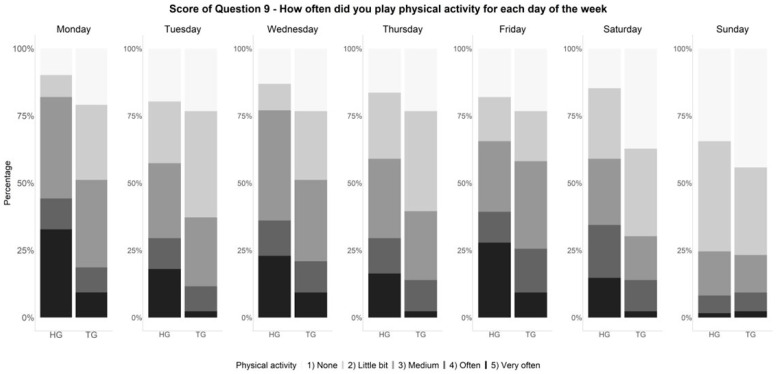
Details of the score of Question 9 of the PAQs questionnaire (frequency of exercise on different days of the week). HG: Healthy group; TG: Transplant group.

Finally, we investigated if there were differences between groups in the score of question 9 in which patients were asked about how often they exercised on different days of the week. Significant differences were observed only on Monday (*p* = 0.05) and on Saturday (*p* = 0.02), as shown in Figure 2.

## 4. Discussion

The present study aimed to investigate if there were differences in lifestyle between a group of healthy children and a group of children who received a solid organ transplant (kidney or liver). It is important to underline that this study was conducted when all school and recreational activities were fully accessible and it was possible to practice indoor and outdoor activities, without any form of restriction due to the COVID-19 pandemic.

Overall, the study population, regardless of their health status, did not exhibit high levels of PA. The average score of PAQs was medium-low in both groups HG and TG (2.69 ± 0.65 vs. 2.42 ± 0.88) with no significative differences. Healthy children did not practice more PA than transplant recipients and we would have expected to see a more marked difference between the two groups. This suggests that children engage in low levels of physical activity regardless of their health status as it happens in the adult population [26].

Comparing the results of our study with existing literature, the average PA score of transplant patients seems similar to what has been observed in other studies, with mean values of 2.2 (1.7–2.9) and 2.8 ± 0.8 [27,28]. On the other hand, healthy children in this study had an average score close to the cut-off value defined for a healthy population as “sufficiently active” by Voss and colleagues (>2.9 for boys and >2.7 for girls) [29]. However, the average score is below the value of 3 which was considered as the threshold value to define as “high activity” by Chen and colleagues [30]. Even healthy children who participated in competitive sports did not reach this threshold (2.82 ± 0.59), indicating a concerning scenario.

These results suggest the need for both populations to engage in more physical activity. Transplanted children, without contraindications, should strive to achieve “sufficiently active” levels of PA to benefit their health, as PA has been shown to be effective in improving outcomes in this population [27,28]. On the other hand, healthy children should be encouraged to practice a greater amount of PA to avoid the negative consequences of sedentariness on their health, such as weight gain [15], poor posture, or increased sedentary sitting time [31].

Adherence to WHO recommendations for physical activity is crucial for both populations to avoid the risks associated with a sedentary lifestyle [16,32]. This is particularly important for transplant recipients, who may be more susceptible to cardiovascular complications due to their underlying conditions or pharmacological therapies [33,34]. Recent evidence showed that poor exercise capacity also affects mortality on the waiting list and in the post-transplant period after all types of solid organ transplantation. Therefore, having good cardio-respiratory fitness before facing a transplant is a new goal to achieve to improve survival after surgery. In recent years some studies have been conducted which demonstrate the importance of practicing physical activity even before transplantation. A meta-analysis of 24 studies showed that physical exercise in this setting leads to significant improvements in exercise capacity (VO_2_ peak), physical performance, and quality of life [22]. Physical inactivity negatively impacts the physical, mental, and social well-being of transplanted patients, as chronic illness can lead to isolation and various negative consequences for their health [22].

To address sedentary lifestyles, introducing “active breaks” during classroom activities could be a first step toward countering sedentary behavior during school hours. Such interventions have been shown to positively impact attention and academic performance in children [35].

Significant differences between the HG vs TG groups were observed in the mean score obtained on question 2, which assessed the practice of physical education lessons at school. Healthy children scored higher in this aspect, highlighting the need to actively involve transplanted children in physical education lessons. These lessons can provide an opportunity for physical exercise without additional costs, promoting inclusion and socialization during school hours.

Further significant differences were observed in the mean scores of question 8, which provided a general impression of physical activity in the last 7 days. A significant difference was found between HG and TG, as well as between competitive and non-competitive healthy children. Although this response is subjective, it motivates the need to encourage transplanted children to engage in more physical activity, fostering their belief in their own ability to be physically active and increasing their confidence.

Regarding question 9, which assessed exercise practice on different days of the week, significant differences between HG and TG were observed only on two days (Monday and Saturday). This suggests that, in general, both groups practiced a similar amount of exercise. However, evidence from a WHO survey [36] shows that in Italy, the prevalence of sufficient PA levels in healthy children and adolescents decreases with increasing age, indicating a need for increased PA across both populations.

At the same time, reports show that physical inactivity is highly prevalent after solid transplant among young patients who should follow the same recommendations [16,32] as healthy children. So, both populations should be advised to play a greater amount of structured and unstructured sporting activities to be practiced both individually and in groups.

The study utilized self-reported PAQs questionnaires, which were chosen for their ease of administration and cost-effectiveness. These questionnaires have been widely used in research involving both healthy and transplanted children, demonstrating acceptable psychometric properties [23]. These questionnaires demonstrated an acceptable-to-good internal consistency, test-retest reliability, and sensitivity to detect gender differences [24,29,37,38,39]. However, a limitation of the study is that self-reported questionnaires provide a subjective measure of physical activity, and it would be beneficial to combine them with objective evaluation methods such as pedometers or wearable devices to more accurately quantify physical activity levels.

The strength of this study is its use of the same simple tool for assessing health in both healthy and transplanted children. This questionnaire allowed for a quick investigation of various aspects of children’s daily lives related to their health and provided appropriate advice and guidance on the importance of regular exercise.

Today a lack of international Guidelines on PA in transplanted patients denies us the possibility of being able to give definite indications on the amount, intensity, and frequency of physical exercise suitable for this type of patient. However, more and more groups of researchers are working to draw up guidelines that will be valid at an international level. The prescription of individualized intervention with an exercise program based on patients’ characteristics, medical history, and individual needs, might induce several positive effects on health and quality of life and might protect the body against the stress related to pharmacologic treatments [40]. It is necessary to work globally to reduce physical inactivity but greater attention must be paid to special categories of children such as transplant recipients. It is essential to inform the children and their families about the benefits of physical exercise participation on health and quality of life. It is also important to encourage and support the practice of PA both at school and in free time, starting as early as childhood. This will allow for a better process of social inclusion of these children, ensuring a general improvement in psycho-physical well-being and cardiovascular health.

Although the results align with previously published data, some important considerations emerge. As proposed by the WHO, children should perform at least an average of 60 min/day of moderate to vigorous PA including sports and leisure activity across the week [16]. Lupo and colleagues proved, in their study, a cut-off value of 2.75 on PAQs to identify “active children” who practice almost 60 min/day of moderate to vigorous PA [41]. In this study, neither healthy children nor those who had received a transplant achieved these values. So, it is necessary to prompt both to practice more PA.

The lack of physical activity in the school environment among transplant patients may be related to teachers’ concerns about managing children with pathologic conditions. This attitude may stem from a cultural exclusion of this population from participating in physical activities. Additionally, pediatric transplant patients often face limitations in engaging in common play and motor activities during the early years of life, requiring more focus on basic motor patterns than their healthy peers. It must also be taken into consideration that some conditions such as fatigue, pain, and poor mental health can represent a barrier that limits participation in physical activity. An adapted physical activity must take these requirements into consideration while trying to minimize their impact on exercise practice and daily life.

In the future, an integrative approach alongside conventional post-transplant treatments, including adapted physical exercise programs, should be considered. This approach could involve various professionals such as surgeons, sports medicine specialists, and kinesiologists to overcome barriers limiting participation in sports activities for these children. It is important to provide all children with opportunities to practice a variety of physical activities that are enjoyable and safe. At the same time, it is important to encourage them to participate in activities that are appropriate for their age and ability. Moreover, all the new recommendations indicated by Leunis et al. [22] such as encouragement and support for family members and friends, education of teachers about the role of exercise and how to practice safely, a greater involvement of children through activities promoted through social networks may contribute to increase physical activity for transplantation recipients reducing physical inactivity and consequently all negative effects on their health. Patients and healthcare providers need to be informed about why, how, when, how often, and with who to be physically activity in a safe way. At the same time the presence of associations or online aggregation groups that bring children from different places into contact, encourages participation in sports as entertainment rather than competition. Even simple and fun activities can be a source of well-being for these patients and should therefore be recommended. It is known that moderate physical activity for just 15 min a day results in a 14% reduction in all-cause mortality compared with inactive subjects [22].

## 5. Conclusions

This study showed that there were no significant differences in lifestyle between healthy children and children who received a solid organ transplant (kidney or liver). Both the Healthy Group and the Transplanted Group did not practice a high level of physical activity when there were no contraindications. Contrary to what we expected, the healthy population did not engage in more physical exercise than the population of children who had received an organ transplant.

This means that all children are exposed to an increased risk of being sedentary and probably they are not aware of their condition. As evidenced by our data, many of them think they have a fairly active life and are not exposed to the complications of a sedentary lifestyle.

Prescribing physical activity should be incorporated into long-term healthcare programs for transplant recipients to improve their cardiovascular health and both length and quality of life. Additionally, it is important to encourage healthy children to engage in more physical activity to experience a greater number of health benefits.

## Figures and Tables

**Table 1:** Demographic characteristics of the study population.

	Gender (n = 104)		Age	BMI (kg/m^2^)
	Male	Female	(Mean ± SD)	(Mean ± SD)
Healthy group	35	26	12.75 ± 2.8	20.1 ± 3.65
Transplant Group	18	25	12.86 ± 3.5	19.47 ± 3.46
Total	53	51	12.8 ± 3.16	19.84 ± 3.57

BMI: body mass index; SD: standard deviation.

**Table 2:** The final score of the PAQs questionnaire.

Population	Score of PAQs Questionnaire (Mean ± SD)	*p*-Value
Healthy group	2.69 ± 0.65	0.08
Transplant Group	2.42 ± 0.88	
Competitive Group	2.82 ± 0.59	0.09
Not Competitive Group	2.53 ± 0.70	
Tx Liver	2.51 ± 0.91	0.21
Tx Kidney	2.16 ± 0.75	

PAQ: Physical Activity Questionnaire; SD: standard deviation; Tx: transplant.

**Table 3:** Details of the score of Question 2 of the PAQs questionnaire (physical education lessons at school).

Population	Score of Question 2 of the PAQs Questionnaire (Mean ± SD)	*p*-Value
Healthy group	3.66 ± 1.58	0.05 *
Transplant Group	3.05 ± 1.59	
Competitive Group	3.97 ± 1.33	0.08
Non-Competitive Group	3.26 ± 1.58	
Tx Liver	3.12 ± 1.70	0.53
Tx Kidney	2.82 ± 1.25	

PAQ: Physical Activity Questionnaire; SD standard deviation; Tx: transplant; * *p* ≤ 0.05.

**Table 4:** Details of the score of Question 8 of the PAQs questionnaire (general impression of physical activity in the last 7 days).

Population	Score of Question 8 of the PAQs Questionnaire (Mean ± SD)	*p*-Value
Healthy group	3.13 ± 1.02	0.02 *
Transplant Group	2.63 ± 1.22	
Competitive Group	3.47 ± 0.75	0.005 **
Non-Competitive Group	2.7 ± 1.17	
Tx Liver	2.75 ± 1.22	0.26
Tx Kidney	2.27 ± 1.19	

PAQ: Physical Activity Questionnaire; SD standard deviation; Tx: transplant; * *p* ≤ 0.05; ** *p* ≤ 0.005.

## Data Availability

Not applicable.

## Appendix A

**Table A1:** Details of PAQ-C and PAQ-A questionnaires.

Question	Response Scored 1	Response Scored 2	Response Scored 3	Response Scored 4	Response Scored 5
1. Physical activity in your spare time: Have you done any of the following activities in the past 7 days (last week)? If yes, how many times?	No	1–2	3–4	5–6	7 times or more
2. In the last 7 days, during your physical education (PE) classes, how often were you very active (playing hard, running, jumping, throwing)?	I don’t do PE	Hardly ever	Sometimes	Quite often	Always
3. In the last 7 days, what did you do most of the times at the access?	Sat down (talking, reading, doing schoolwork)	Stood around or walked around	Ran or played a little bit	Ran around and played quite a bit	Ran and played hard most of the time
4. In the last 7 days, what did you normally do at lunch (besides eating lunch)?	Sat down (talking, reading, doing schoolwork)	Stood around or walked around	Ran or played a little bit	Ran around and played quite a bit	Ran and played hard most of the time
5. In the last 7 days, on how many days right after school, did you do sports, dance, or play games in which you were very active?	None	1 time last week	2 or 3 times last week	4 times last week	5 times last week
6. In the last 7 days, on how many evenings did you do sports, dance, or play games in which you were very active?	None	1 time last week	2 or 3 times last week	4 or 5 last week	6 or 7 times last week
7. On the last weekend, how many times did you do sports, dance, or play games in which you were very active?	None	1 time	2 or 3 times	4 or 5 times	6 or more times
8. Which one of the following describes you best for the last 7 days?	All or most of my free time was spent doing things that involve little physical effort	I sometimes (1–2 times last week) did physical things in my free time (e.g., played sports, went running, swimming, bike riding, did aerobics)	I often (3–4 times last week) did physical things in my free time	I quite often (5–6 times last week) did physical things in my free time	I very often (7 or more times last week) did physical things in my free time
9. Mark how often you did physical activity (like playing sports, games, doing dance, or any other physical activity) for each day last week	None	Little bit	Medium	Often	Very often
10. Were you sick last week, or did anything prevent you from doing your normal physical activities?	Non-evaluable score	Non-evaluable score	Non-evaluable score	Non-evaluable score	Non-evaluable score
